# Feasibility and acceptability of the Promoting Pupils’ Physical Literacy (3PL) intervention and its effectiveness research design: A study protocol

**DOI:** 10.1371/journal.pone.0294916

**Published:** 2024-01-05

**Authors:** Thea Toft Amholt, Mette Kurtzhals, Paulina Sander Melby, Anna Stage, Johann Issartel, Wesley O’Brien, Sarahjane Belton, Mads Bølling, Glen Nielsen, Peter Bentsen, Peter Elsborg

**Affiliations:** 1 Center for Clinical Research and Prevention, Copenhagen University Hospital–Bispebjerg and Frederiksberg, the Capital Region of Denmark, Copenhagen, Denmark; 2 Department of Nutrition, Exercise and Sports, University of Copenhagen, Copenhagen, Denmark; 3 Department of Sports Science and Clinical Biomechanics, University of Southern Denmark, Odense, Denmark; 4 School of Health and Human Performance, Faculty of Science and Health, Dublin City University, Dublin, Ireland; 5 Physical Education, Sports Studies and Arts Programme, School of Education, University College Cork, Cork, Ireland; 6 Research Centre for Pedagogy and Bildung, Program on Outdoor Pedagogy, VIA University College, Aarhus, Denmark; 7 Department of Geoscience and Natural Resource Management, University of Copenhagen, Copenhagen, Denmark; PLOS: Public Library of Science, UNITED KINGDOM

## Abstract

Research has shown that physical activity (PA) is important for health throughout the lifespan. Therefore, it is important that children develop the individual prerequisites that enable participation in PA throughout life. The theoretical concept physical literacy (PL) and the research field of PL has described such personal competences and traits. However, to promote PL among children and lifelong PA, there is a demand for more high-quality interventions to be developed and tested. When targeting children, schools are an important setting. Despite the possibility of promoting PL during PE lessons, few well-tested interventions have been developed. In this study, we therefore aim to context adapt and feasibility test an already existing and promising PL intervention to a Danish school context. The ADAPT and MRC guidelines were followed to adapt the Promoting Pupils Physical Literacy (3PL) intervention. Through workshops with stakeholders, the intervention was adapted to fit Danish 4^th^ and 5^th^ graders. Four Danish schools were recruited in a wait list design. The feasibility and acceptability of both the intervention and the effect study design will be investigated. To investigate the intervention, weekly questionnaires, observations, and interviews will be conducted during the intervention period. The feasibility of the effect study design will be investigated by collecting baseline and endline data on pupils’ PL and daily PA as well as parents’ socioeconomic status. Expected outcomes include a TIDieR checklist, a revised, feasible, and acceptable intervention, and an effect study design protocol. This will contribute to important steps in the direction of making PL interventions more accessible for practice. Valid testing of intervention effectiveness enables stakeholders to make informed decisions grounded in evidence. This will strengthen the possibilities of a successful outcome and for a PL intervention that is more accessible for practice, which is important for scale up.

## Background

The increasing prevalence of non-communicable diseases (NCDs) and obesity, along with their significant societal costs, is a growing global issue. Physical inactivity plays a crucial role as a primary risk factor contributing to the development of both obesity and NCDs [1–4]. Despite this fact, recent assessments of PA have highlighted a concerning issue–regardless of age, cultural backgrounds, and gender–communities worldwide are struggling to adhere to international and national PA guidelines [1, 5]. The life-course approach to health promotion highlights the benefits of targeting children and youth when aiming for lasting behavioral changes to enhance health, particularly concerning increased engagement in PA [6]. From a life-course perspective, prevention and health promotion interventions should shift from exclusively targeting factors and behaviors directly predicting adult health, such as PA, toward addressing the foundational elements of for example a physically active lifestyle over the life-course, in order to address the underlying causes behind the causes of health [7, 8].

When it comes to interventions aimed at promoting PA and it’s benefits for health this means that rather than solely concentrating on increasing PA levels in terms of quantity and intensity, the focus should be on nurturing the prerequisites necessary for participating and engagement in PA across various contexts throughout one’s lifetime [9, 10].

The concept of physical literacy (PL) provides a framework for conceptualizing and describing these essential prerequisites. PL introduces a perspective to PA behavior with the aim to foster enduring shifts in children’s engagement in PA across the different facets of their daily lives. PL is a multidimensional and comprehensive concept, acknowledged as a fundamental determinant for PA participation throughout one’s life [7]—a key ‘cause of the causes’.

In 2010, Margaret Whitehead introduced the concept of PL, with the following definition: “*Physical literacy is the motivation*, *confidence*, *physical competence*, *knowledge and understanding to value and take responsibility for engagement in physical activities for life*” [11]. In this way, PL encompasses important personal attributes and essential prerequisites required for engaging in PA across various settings and across the lifespan.

### PL interventions

Research on how to promote PL is still in its infancy, with a limited number of studies employing experimental designs. Thus, there is a limited number of evidence-based examples showcasing deliberate approaches to promote PL comprehensively and evaluating effects on both PL and levels of PA [12]. This is an evident requirement to advance our understanding of *how* to enhance children’s PL.

Schools are widely recognized as a pivotal environment for promotion of PA among children [13], and physical education (PE) plays an important role in nurturing a child’s development of PL [11, 14–17]. Systematic reviews and meta-analyses have reported mixed results regarding the impact of PA interventions on well-being [18, 19]. One potential explanation for these discrepancies could be the impact of how PA is delivered [20], which is a pivotal aspect highlighted in the theory of the mechanisms of PL [11].

In the beginning of 2021, the authors of this paper conducted a rapid review of interventions related to PL. Throughout the review process, factors associated with effective PL interventions, as mentioned above, were considered. Of the reviewed interventions, the Youth-Physical Activity Towards Health (Y-PATH) program emerged as the most extensively developed and evaluated. Y-PATH is grounded in the holistic and multidimensional theory of PL and designed for first to third secondary school students (i.e., adolescents aged 12–15 years) [21, 22].

The primary goal of the Y-PATH intervention is to enhance the PA levels of adolescents by addressing the various domains of PL during PE lessons, including both affective, physical, and cognitive aspects. The effectiveness of the Y-PATH intervention has been rigorously tested in Ireland, yielding positive results by enhancing levels of moderate to high intensity PA and improving fundamental movement skills [23–25].

To work with and spread the positive effects of PL interventions such as Y-PATH calls for an investigation of the possibility to translate and adapt interventions across countries and contexts. The demand for empirically validated PL interventions that substantiate their efficacy and effectiveness is thus pressing. However, the necessity of testing feasibility and acceptability prior to conducting a Randomized Control Trial (RCT) cannot be overstated [26]. Feasibility studies provide important preliminary investigations of the practicality of the RCT, including the recruitment process, data collection procedures, and potential logistical challenges. This initial assessment allows researchers to identify and rectify potential issues, refine research protocols, and optimize resource allocation [27]. Furthermore, assessing the acceptability of the proposed Y-PATH intervention among the Danish target population is integral to ensuring its effectiveness within this context. By evaluating the acceptability of the Y-PATH intervention, we can adapt and tailor the intervention to better align with the preferences and needs of the participants and the teachers, enhancing its chances of success in general and in the subsequent RCT [26].

Therefore, conducting a feasibility and acceptability study prior to embarking on an RCT of the Y-PATH intervention is important for mitigating the risks associated with resource allocation and for enhancing the likelihood of conducting a meaningful and impactful RCT.

## Methods

The purpose of the present work is to transparently describe and discuss the protocol for adapting the Y-PATH intervention and evaluating the feasibility and acceptability of the Danish version named Promoting Pupils’ Physical Literacy (3PL). The aim of the of this study protocol is twofold:

1. to systematically evaluate and test the feasibility, acceptability, and implementation of the intervention in terms of fidelity, i.e., adherence, dosage, quality of intervention delivery, participant responsiveness, and program differentiation

1. to evaluate the feasibility of the effect study design.

The study builds on an adaption process that takes an iterative, dynamic approach, focusing on the translation and context adaptation of the Y-PATH intervention. The authors confirm that all ongoing and related trials for this intervention are registered. The 3PL study was pre-registered at clinicaltrials.gov (ID NCT05822024), April 2023, version 1 (https://classic.clinicaltrials.gov/ct2/show/NCT05822024). Due to staff change in the middle of the process, the final registration was completed during the recruitment process, April 2023. The initial thoughts and plans for the protocol for the adaptation process of the intervention was pre-registered at Open Science Framework [28]. The first step was to translate and adapt the Y-PATH intervention to a Danish context (described in the section ‘adaptation’). The next step follows a cluster randomized waitlist design. Four schools were sampled and randomly assigned to either a control or an intervention group (described in the section ‘sampling and recruitment’). PE teachers at intervention schools will undergo a four hours in-person and a module-based online course (i.e., four modules of 20 minutes) before the intervention starts. Meanwhile, pupils will complete baseline tests. The evaluation will use a mixed-method approach, as proposed by the Medical Research Council (MRC) guidance for developing complex interventions (described in the section ‘data collection and measurements’) [26].

### Ethics

The 3PL study was pre-registered at clinicaltrials.gov (ID NCT05822024), April 2023, version 1 (https://classic.clinicaltrials.gov/ct2/show/NCT05822024), and the protocol for the adaptation procedure of the Y-PATH intervention was pre-registered at Open Science Framework, December, 2022 [28]. The 3PL study protocol was assessed by the Regional Committee on Health Research Ethics, Capital Region, Denmark, March 2023 (ID: 2106200699). The committee determined that, in accordance with Danish law, the protocol did not qualify for ethics review, as all non-biomedical research projects fall outside the scope of eligibility for ethics assessment. All data and any associated personal information will undergo processing in strict adherence to the Danish Data Protection Act. Prior to enrollment in the study, consent based on written communication will be secured from teacher and parents or legal guardians. Oral communication will be employed when addressing the children. The data collection processes will be conducted in an engaging and inclusive manner that prioritizes the well-being of the children. Attention will be given to the fact that collecting of questionnaire data may divert time from curricular activities; however, the investigation of motor competence and PA measurement aligns with the existing PE curriculum. Children will be provided with the opportunity to decline PA measurements in the event of potential skin irritation and to opt out of participation in other data collection activities if they find them discomforting. To acknowledge their valuable contributions, teachers and classes will receive separate reimbursements of 200 EUR (1500 DKK) for each participating class. Trail data will be made accessible upon request with the authors.

**Fig 1:**
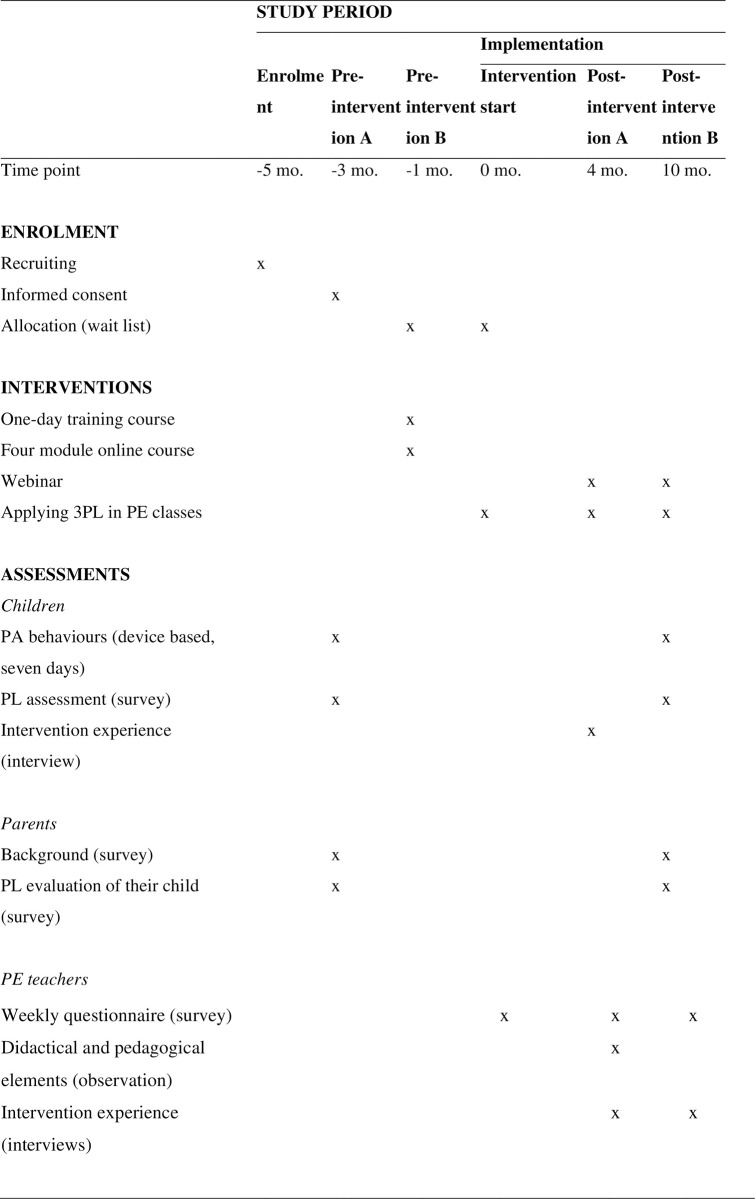
Participant allocation timeline according to SPIRIT.

Fig 1 provides an overview of participant allocation timeline according to SPIRIT.

**Fig 2:**
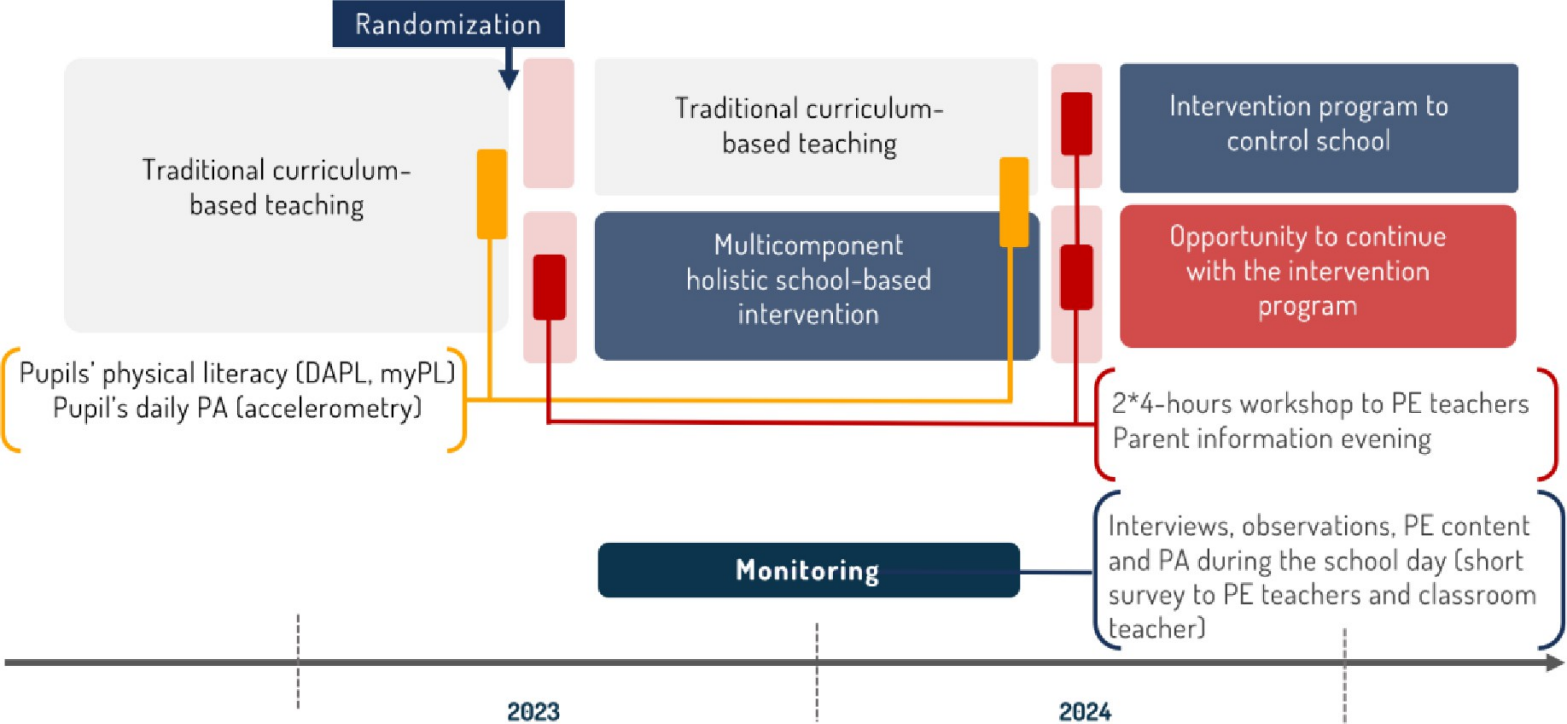
Study design overview and timeline.

An overview of the study design and timeline can be found in Fig 2.

### Theoretical framework

**Fig 3:**
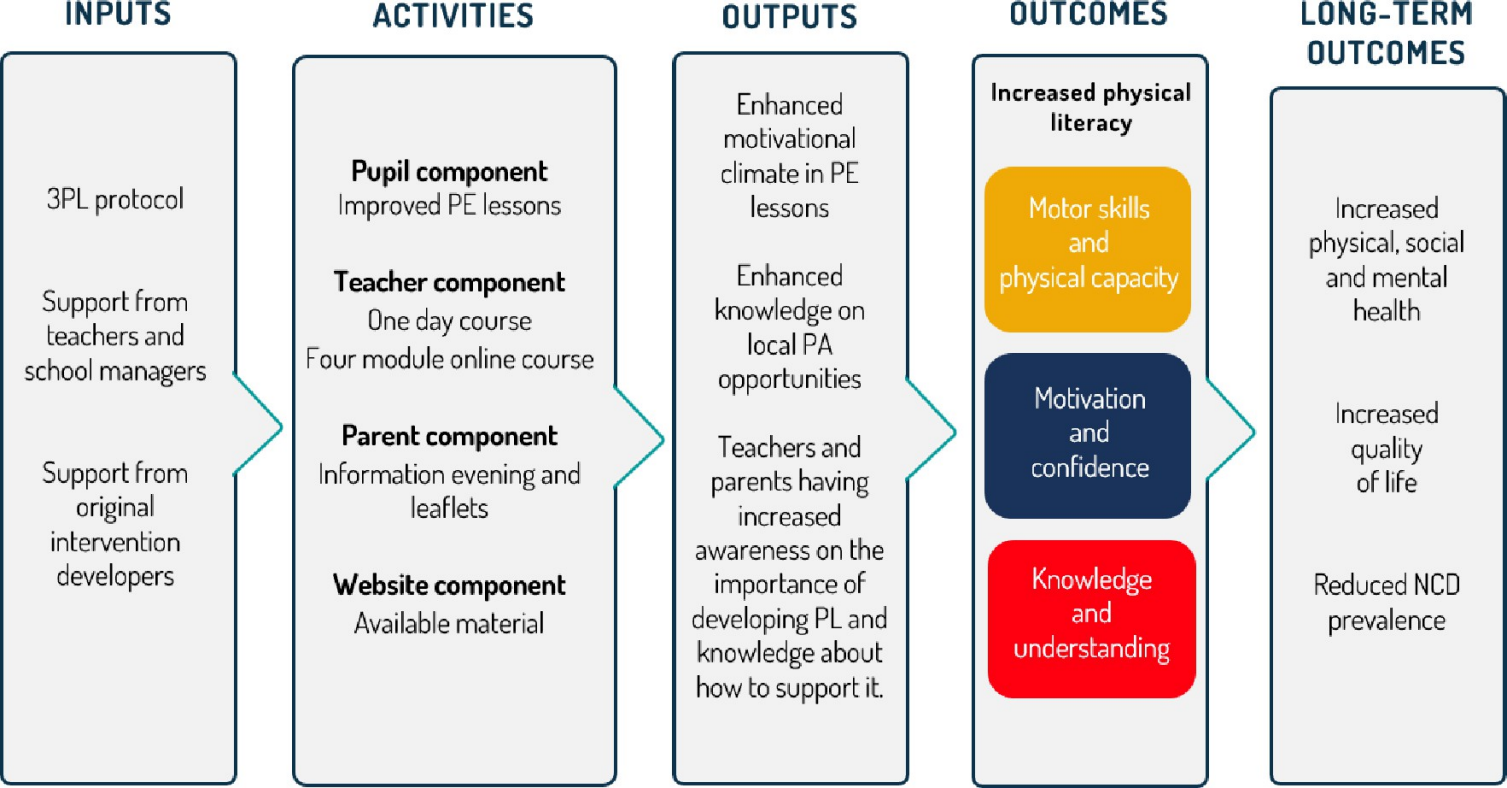
Logic model of the 3PL feasibility study.

The theoretical framework of the 3PL feasibility study is illustrated graphicly in a logic model, Fig 3. The logic model depicts the underlying theory of change and provides an overview on the relationship between 3PL resources, activities, the intended outputs of these components, and the immediate outcomes. Also, the possible long-term impacts of the activities are outlined. It is theorized that the intervention activities including a pupil, teacher, parent, and website component will result in the following outputs; enhanced motivational climate in PE lessons, enhanced knowledge on local PA opportunities, teachers and parents having increased awareness on the importance of developing PL and knowledge about how to support it. This was also found in the original Y-PATH intervention [23, 24]. Immediate outcomes will include increased PL which thus will lead to long-term outcomes such as increased physical, social, and mental health, increased quality of life, and reduced NCD prevalence.

### Setting

The Danish school system will be the primary setting for the intervention. The primary and lower secondary schools in Denmark have the same general curricular structure nationwide. The Danish Parliament legislates the overall aims of education in Danish schools with specific targets for each subject. Yet, the local municipalities, schools, and teachers have so-called ‘freedom of methods’, i.e., a teacher has the autonomy to choose how to achieve the specific targets within their specialization subjects such as PE.

The Danish schools typically have classes ranging from 0 to 9^th^ grade (i.e., 5–16 years of age) divided into three sections: junior 0 to 3^rd^ grades (5–10 yrs.), middle 4^th^ to 6^th^ grades (10–13 yrs.), and senior 7^th^ to 9^th^ grades (13–16 yrs.) [29]. Each class contains a maximum of 28 gender-mixed pupils.

The 3PL intervention will be facilitated within the principles of the ‘add-in’ approach to school health promotion increasing adherence of participants [30], as the intervention supports the delivery of the current national PE curriculum, and thus does not add on any additional workload for PE teachers. The secondary setting for the intervention will be a home setting. That is, parents will be invited to actively participate in the children’s PL journey by interactive evening sessions and online education about PL.

#### Sampling and participants

*Recruitment*. Recruitment and enrollment ran from February 2023 to June 2023. Firstly, information about the project was spread and after reply from Scientific Ethics Committee, March 2023, schools were contacted and offered enrollment. Teachers were recruited via the local school principals and through municipal school directors. Schools were initially contacted via e-mail and in cases of non-responds, contacted via telephone. Online information meetings were held with the interested school principals and relevant teachers. On the schools who chose to participate, the head teachers of the involved classes informed the parents or legal guardians and provided them the written information about the project. In total, four elementary schools were recruited: two from a rural part and two from an urban part of Denmark. In each school, all 4^th^ and 5^th^ grades (ages 9–11) were included. This age group was chosen due to 1) the explicit demand of working with motoric aspects of PE during these years from the Ministry of Education [31] and 2) the possibility for altering PE lessons in middle school in contrast to grade 7^th^ to 9^th^ where PE lessons are more structured and focused on exams. The schools were distributed in two different regions in Denmark within three different municipalities, and were located in areas of varying size, economic background of residents in the municipalities, housing densities, access to sports facilities, and green areas (see Table 1).

**Table 1:** Characteristics of participating schools.

	School 1	School 2	School 3	School 4
Number of participating children	52	90	92	80
4^th^ grade / 5^th^ grade participating	1 / 1	2 / 2	2 / 2	2 / 2
Average income pr. year in municipality (DKK)	206.726	206.726	205.179	220.945
Rural/urban	Rural	Rural	Urban	Urban
Intervention/control	Intervention	Control	Control	Intervention

*Intervention allocation*. The allocation of the four schools was done by block randomization and schools were matched on location (i.e., each group consisted of an urban and rural school). Allocation was performed by the first author on May 8^th^, 2023, and publicly transmitted in an online Teams meeting for everyone interested to join to ensure a transparent process. Immediately after, the enrolled schools and teachers were informed by e-mail whether they were allocated to intervention or control group. A waitlist design will be adopted meaning that two schools will start with the intervention in the school year 2023/24. The two other schools will be enrolled in the intervention in the school year 2024/25.

#### Adaptation process

The aim of the adaptation process was to increase the likelihood of the Y-PATH intervention, here named the Promoting Pupils’ Physical Literacy (3PL) intervention, being acceptable, feasible, engaging, relevant, and further effective and sustainable, within a Danish context. The adaption process was guided by the evidence and consensus informed ADAPT process model and its principles and actions for adapting and transferring an intervention into a new context, outlined by Moore et al. [32]. That is, the following principles and actions were considered before and recurrently during the adaptation process:

- The degree and format of the involvement of stakeholders, including those with expertise in the original intervention as well as those with knowledge of the Danish school context.
- The assessment of and attention to the rationale for the context-adapted intervention and considerations regarding the best possible Danish school fit with focus on the effectiveness, cost-effectiveness, and implementation of the intervention.
- The plans suggested for adaptations as well as choice of adaptions necessary for the intervention to function as intended in terms of change mechanism.
- Future implementation and maintenance of the intervention at a scale.

Throughout the onward adaption process, iterative cycles of modification with stakeholder input will be used to refine the translated intervention content and the adapted materials–to achieve the best fit between the intervention and context.

**Fig 4:**
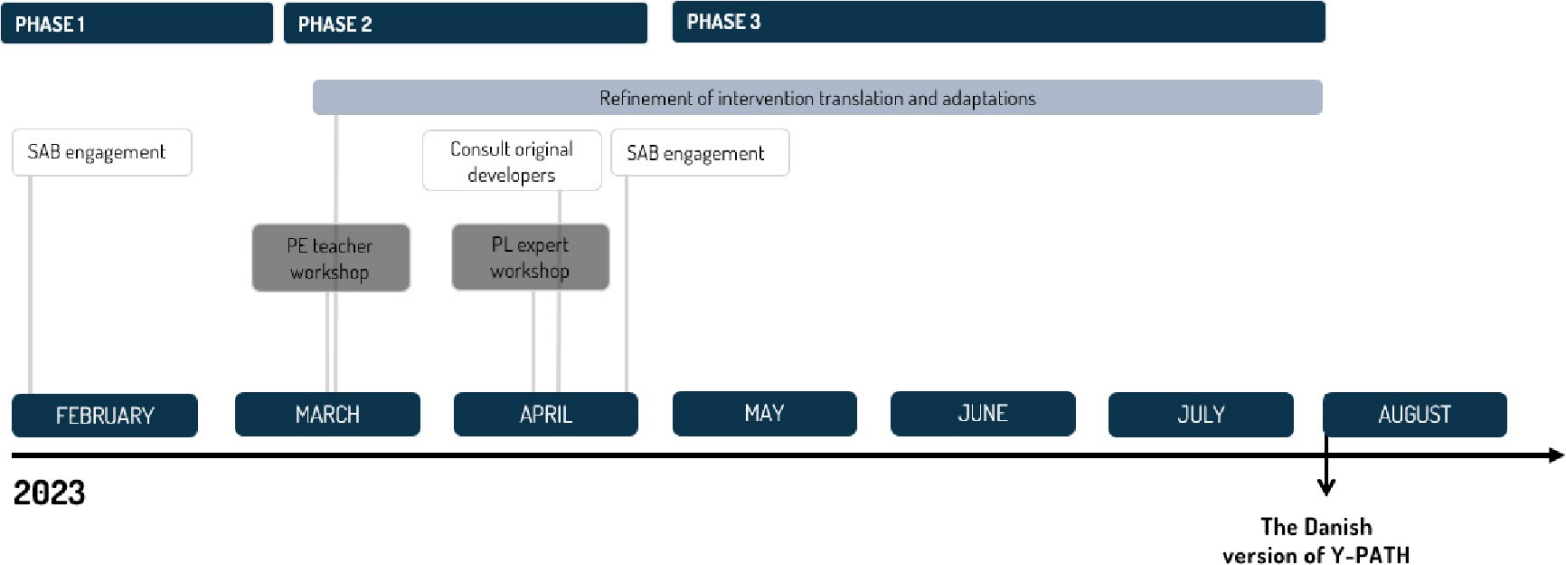
Overview of the adaptation process.

Fig 4 presents an overview of our adaption process, elaborated in detail at Open Science Framework [28]. Firstly, a scientific advisory board (SAB) of national and international experts within the PL intervention research fields were gathered to provide feedback on and discuss the adaptation process and its protocol [28] (i.e., Phase 1 of the adaption process). Secondly, the intervention content and materials were translated, and minor changes were suggested, both regarding content and materials, to adapt the intervention to a Danish context (i.e., Phase 2 of the adaptation process). Subsequently, the materials were evaluated and discussed at two workshops: one with PE teachers–to achieve the best fit with the school context, and another one with PL experts–to attain the change mechanisms revealed in the original intervention. At the workshops, the intervention materials were discussed and altered according to feedback. After the workshops, the original developers of the intervention were consulted to discuss these alterations. Finally, all the feedback was systematically analyzed and used as a foundation for the final refinement of adaptations of the content and materials (i.e., Phase 2 and 3 of the adaptation process).

#### Data collection and measurements

**Fig 5:**
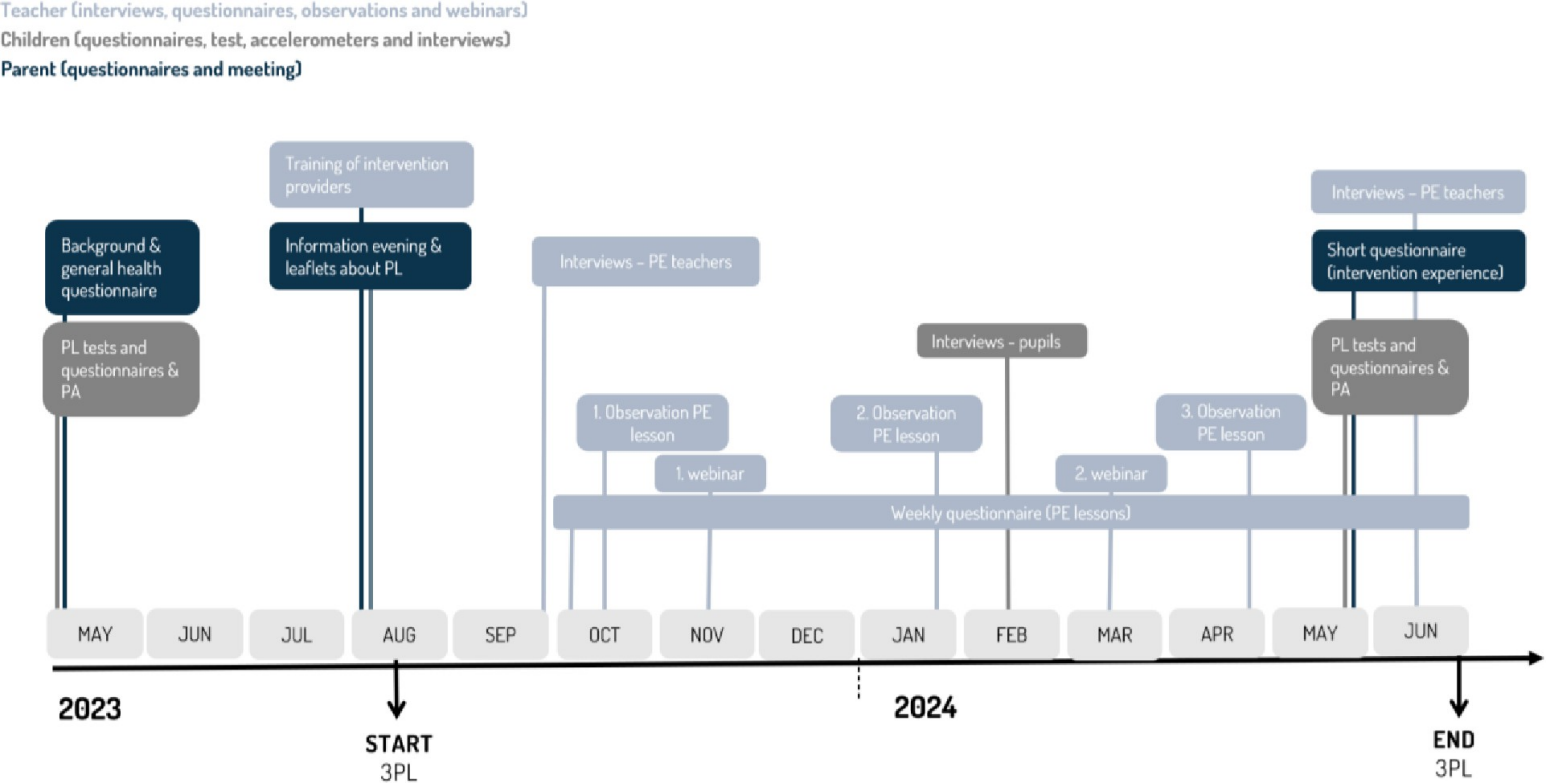
Data sources and overview of data collection.

The measurements will be based on the MRC guidance and Bowen’s et al. (2009) areas of focus for feasibility and acceptability of testing an intervention. Data about feasibility and acceptability will be collected from teachers, parents, and children. Teachers will be invited to interviews, participate in two webinars, receive a weekly questionnaire, and lastly their PE lessons will be observed by researchers. As for the children, their daily PA and PL will be assessed at two time points (at baseline and endline). During the intervention, they will be invited to participate in focus-group interview. Data from the parents will be collected by two questionnaires, one including background information, and the other will involve an assessment of their child’s PL. All data will be uploaded in a secure folder logging all entries. Only members of the project group will have access to the data. No data monitoring committee is therefore needed. Fig 5 illustrates the overview of data sources and collecting time of measurements.

#### Outcome measures, feasibility, and acceptability of the intervention

*Use of intervention materials*. During the intervention school year, PE teachers of the involved classes will receive a short weekly questionnaire consisting of three items: “Did you teach PE this week?”, “Did you use materials from 3PL?”, and “Which materials did you use?”. The questions focus on their use of the intervention materials, to assess the relevance and usefulness of these on a regular basis. In case of no response, a reminder will be sent after three days.

*Pedagogical and didactical elements*. A minimum of eight semi-structured interviews will be carried out to assess the pedagogical and didactical elements of the intervention delivery in terms of intervention adherence, motivation climate in PE lessons, and use of PL guided activities. The interview guides will be inspired by the theoretical framework of acceptability in healthcare interventions [33]. The interviews will be conducted by a facilitator and a note-taker. The facilitator’s role will be to guide the focus-group, stimulate interaction among participants toward the theme(s), oversee group discussion, and encourage everyone to contribute and respond, while the note taker will keep a record of the discussion as it evolves to add details for instances where recording is not audible. All interviews will be digitally recorded and transcribed verbatim, de-identified, and uploaded into NVivo. Furthermore, a minimum of eight semi-structured observations will be carried out to validate the interview statement and assess pedagogical and didactical environment [34].

*Quality of intervention delivery*. The quality of the delivery of the intervention, local variations, and acceptability of the intervention will also be obtained through semi-structured focus-group interviews with children and individual interviews with teachers as described above [35, 36].

#### Outcome measures, feasibility, and acceptability of the effect study design

*Interviews with teachers and children*. Experiences with the design of the study including recruitment, randomization process, and data collection procedure will be assessed through semi-structured focus-group interviews with children and individual interviews with teachers [35, 36]. These interviews will also be used as an outcome measure for feasibility and acceptability of the effect study design.

*Physical literacy*. PL will be measured with three different measures: 1) Danish Assessment of Physical Literacy (DAPL), 2) My Physical Literacy (MyPL), and 3) My Physical Literacy parent (MyPL-parent). The three measures are different in terms of resource demand, theoretical foundation, and mode of delivery. In this study, the three different measures will be tested to enable a comparison of the feasibility, acceptability, and relevance of the measures. The measures are described below.

*Danish Assessment of Physical Literacy (DAPL)*. DAPL [10] is an adaptation of the Canadian Assessment of Physical Literacy second edition [37, 38]. The affective domain of PL will be assessed through the utilization of four validated questionnaire-based scales, which will gauge the participants’ levels of autonomous motivation and confidence for physical activities. The cognitive domain will be evaluated through questions designed to measure the participants’ knowledge and comprehension regarding PA. The physical domain will be assessed through a combination of a strength, endurance, and motor competence tests. The scores will be standardized in point distributions as follows: the affective domain will range from 0 to 30 points, the physical domain from 0 to 30 points, and the cognitive domain from 0 to 10 points. Furthermore, a total PL score for each child will be derived by summing the respective scores, yielding a range of 0 to 70 points. The assessment of PL will be completed in two PE lessons one week apart.

*My-PL*. The MyPL questionnaire measures PL in specific contexts in order to take into account that PL can differ across different social and physical environments for PA. The first edition of the MyPL questionnaire has previously been validated and used in other studies [39]. The second edition used in this study consists of five subscales: 1) PL for ball- and running-based activities, 2) PL for playground-based activities, 3) PL for gymnastic-based activities, 4) PL for skate-based activities, and 5) PL for water-based activities. Each subscale consists of environment specific items related to the affective domain, the physical domain, and the cognitive domain respectively. The questionnaire is video assisted, so that the specific environments and contexts are explained with voiceover to the children and the items are read aloud.

*MyPL-parent*. The MyPL questionnaire was adapted to assess parent reported PL of their children. MyPL-parent has the same overall structure as the child reported version of PL. It assesses the three PL domains (i.e., the affective, physical, and cognitive) specific to the same five environments described above. MyPL-parent will be distributed to the parents via the same text message that thanks the parents for their children’s participation on the day of the second PE lesson. In case of no response, a reminder will be sent within three days.

*Physical activity*. PA behaviors will be measured over one week i.e., seven consecutive days. Axivity AX3 accelerometers (23 x 32.5 x 7.6 mm.) will be attached to the left thigh. In the first PE lesson, approximately five children at a time will be taken to a room close to the classroom to have accelerometers attached. A male researcher manages the male participants, and a female researcher manages the female participants. By turn, the Axivity AX3 accelerometer will be fixed directly to the skin with Fixomull (BSN Medical), adhesive hair-set tape (3M, USA) on the medial front of the thigh midway between the hip and the knee joints with the positive x-axis pointing downwards. The procedure follows the recommendations of Petersen and colleagues [40]. In case of accidental removal of the accelerometer in the seven-day period, the children and their parents will be instructed to put the accelerometer back on using extra tape. After one week, the researchers will collect the accelerometers in the PE lesson.

*Socio economic status*. Socio-economic status in terms of the parents or legal guardian’s education level and occupational status will be assessed using the Occupational Social Class Measurement (OSCM). The OSCM will be administered to the children’s parents or legal guardians in a text message sent on the day of the first data collection take place. In case of no response, a reminder will be sent within three days.

## Analyses

### Feasibility and acceptability of the intervention

The analyses of feasibility and acceptability of the intervention will focus on key aspects of adhering to the intervention and delivering the intervention with high quality. The weekly teacher questionnaires will be analyzed with descriptive statistics investigating the overall frequency of use of different intervention materials and whether patterns of use of materials change during the school year. The observations and interviews will be analyzed using thematic analysis to systematically categorize factors affecting feasibility and acceptability of the intervention [41]. The thematic analysis will be a mix of a bottom-up and top-down approach. Emerging themes will be categorized and summed up and the framework for assessing acceptability in health care interventions, developed by Sekhon and colleagues [33], will be used to explore seven aspects of acceptability including affective attitude, burden, ethicality, intervention coherence, opportunity costs, perceived effectiveness, and self-efficacy [33].

### Feasibility and acceptability of the effect study design

The analyses of the feasibility and acceptability of the effect study research design will focus on the key parameters necessary for conducting a future trial. All interviews will be analyzed using thematic analysis to systematically categorized factors related to the effect study design [41]. Most of the statistical analyses will be descriptive in nature. Recruitment attrition, questionnaire response rates, and other assessment attrition for both children and their parents will be descriptively summarized. This will be used to evaluate assessment fidelity, acceptability, and relevance in a future trial.

The three measures of PL (i.e., DAPL, MyPL, and MyPL-parent) will be used to investigate within-subject differences between baseline and follow-up measures in both the intervention and control group. This will be done by conducting linear mixed models with the PL measures as dependent variables and condition (i.e., intervention and control) as independent variables. The models will be adjusted for age, gender, and socio-economic status as well as the clustered nature of the collected data (children in classes in schools). Because of the small sample size, these analyses will be interpreted with caution and will mainly inform power calculations providing insights into sample size requirements for a future trial.

## Discussion

### Expected outcomes

A Template for Intervention, Description, and Replication (TIDieR) checklist [42] will be an essential outcome to comprehensively report the details of a revised, feasible, and acceptable version of the 3PL intervention. Among the TIDieR checklist components, particular attention is given to the contextualization of the modified intervention procedures and materials to align with Danish PA and sport culture, as well as conforming to the regulations outlined in the National Danish PE curriculum [31] and organization of PE lessons. In addition, all intervention materials will be made accessible. Besides outlining the intervention, an account of the intervention costs encompassing aspects such as training, materials, implementation, and evaluation will be documented throughout the feasibility study. These cost details will be coalesced with the TIDieR checklist for comprehensive reporting.

As an additional outcome measure, the feasibility of the research design will be monitored and reported. This assessment will encompass the evaluation of recruitment procedures, materials, randomization processes, and the application of measures and instruments within the school and leisure context. Based on the findings, a comprehensive design for assessing the effectiveness of the 3PL intervention will be proposed.

### Perspectives

As described in the introduction, many studies indicate that PL is important for children’s health and well-being. Many countries now regard PL as a pivotal element in promoting the health and well-being of their populations [43–45]. This widespread embrace of PL can be attributed to its comprehensive and holistic approach, which integrates various disciplinary perspectives [46]. This perspective on PA behavior, becomes imperative in light of the limited long-term impacts found of interventions aimed at increasing the frequency and intensity of PA among children and adolescents [47, 48]. In addition to the small effect sizes of school-based PA interventions, research has highlighted that PA, as a health behavior, is contingent on context and should not be regarded as an inherent trait [49, 50]. This contextual nature may explain why interventions primarily focusing on increasing PA often yield minimal or no long-term effects [47, 48].

Cross-sectional studies have described predictors of PL, which provides a solid foundation for developing interventions that have the potential to promote PL among children. However, few studies have developed and tested the effectiveness of such PL-promoting interventions. With the 3PL intervention, we aim to test and adapt a feasible intervention as well as develop a feasible effect study design. Such studies contribute with important steps in the direction of making PL interventions more accessible for practice.

It is recommended that the school-based health promotion strategies should be underpinned by theoretical frameworks for behavior change and further engage the entire school community, including teachers, school leaders, and parents [51, 52]. It is vital for school-based interventions to align with the core activities of the school rather than being perceived as mere additions to the existing curriculum and extracurricular activities [30]. Interestingly, multicomponent interventions that encompass both curricular and non-curricular aspects of a school day (such as physical education lessons, school recess, and after-school leisure time) have demonstrated the most promising results at increasing PA levels among children and adolescents [53, 54]. A recent meta-analysis and review indicated that interventions rooted in the theoretical framework of PL are indeed effective in promoting PL-related outcomes [12]. The authors note that this conclusion was made despite some limitations related to publication bias. In light of these findings, it becomes evident that health promotion strategies founded on a PL framework holds a crucial promise for practical applications within the realm of health enhancing PA. It has been suggested, that ‘effective’ PL interventions must be designed with the primary goal of promoting all of its core domains i.e., the affective, physical, and cognitive domains. The focus on the multidimensional nature of PL, ensures that the effects of the activities are mutually reinforcing [55–57]. Furthermore, the 3PL intervention will focus on tapping in to the current circumstances in Danish schools and act as an ‘add-in’ rather than an ‘add-on’ to the curriculum [30]. This strengthens the possibilities of a successful outcome of the intervention.

The use of a randomized wait list design will minimize any influences on effect measures used to evaluate the feasibility of the effect study design. RCTs of PL interventions are needed to provide the most robust scientific evidence on whether interventions effectively enhance PL for its target groups [58]. The execution of studies using randomization with sufficient statistical power demands significant resources, including time, funding, and human capital. Before embarking on such a rigorous study design, it is imperative to ascertain the feasibility of the proposed research methods and the acceptability of the intervention within the target population [26]. By addressing these concerns beforehand, the likelihood of successful RCT execution is markedly increased, thus safeguarding the valuable investments of both time and funding [59]. Valid testing of intervention effectiveness enables stakeholders in education, public health, and policymaking to make informed decisions grounded in evidence. It also enables the refinement of health promotion intervention strategies and the adaptation of pedagogical approaches, further augmenting their effectiveness.

## Supporting information

S1 Checklist SPIRIT 2013 checklist: Recommended items to address in a clinical trial protocol and related documents*. (DOC)

S1 File (DOCX)

## List of abbreviations

DKK: Danish currency
EUR: Euro currency
MRC: Medical Research Council
NCD: noncommunicable diseases
PA: physical activity
PL: physical literacy
RCT: Randomized Control Trial
SPIRIT: Standard Protocol Items: Recommendations for Interventional Trials
Y-PATH: Youth Physical Activity Towards Health
3PL: Promoting Pupils’ Physical Literacy
