# Supplementary material for: Feasibility and acceptability of the Promoting Pupils’ Physical Literacy (3PL) intervention and its effectiveness research design: A study protocol
Source: PLoS One. 2024 Jan 5;19(1):e0294916. doi: 10.1371/journal.pone.0294916 (PMC10769070; doi:10.1371/journal.pone.0294916)
Supplement: S1 File — (DOCX) [file pone.0294916.s002.docx]

1. **Scientific title**

Promoting Pupils’ Physical Literacy (3PL) (Danish: Ægte, sjov bevægelse)

Mette Kurtzhals^1,2^, Paulina Melby^3^, Peter Elsborg^1^, Glen Nielsen^2^, Thea Toft Amholt^2^ and Peter Bentsen^1,4^

^1^ Center for Clinical Research and Prevention, Copenhagen University Hospital – Bispebjerg and Frederiksberg, Nordre Fasanvej 57, 2000 Frederiksberg, Denmark
^2^ Department of Nutrition, Exercise and Sports, University of Copenhagen, Copenhagen N, Denmark
^3^ Department of Sports Science and Clinical Biomechanics, University of Southern Denmark, Campusvej 55, 5230 Odense, Denmark
 ^4^ Department of Geoscience and Natural Resource Management, University of Copenhagen, Frederiksberg, Denmark

**Project leader**

Peter Bentsen, Center Director,Center for Clinical Research and Prevention (CCRP), PhD., Affiliated Professor
(e-mail: [peter.bentsen@regionh.dk](mailto:peter.bentsen@regionh.dk)). Nordre Fasanvej 57, Hovedvejen, entrance 5 (building 14), 2000 Frederiksberg, Denmark

**2. Project aims**

**a.** The overall objective is to investigate the feasibility and acceptability of the theory-based and internationally tested intervention, Youth-Physical Activity Towards Health (Y-PATH), within a Danish school context, informed by the Medical Research Council (MRC) framework and The Common Guidelines for Education Research and Development. The goal is that a revised and adapted and feasible intervention protocol, that aims to increase pupils’ PL, will be ready for effectiveness testing by the end of this project.

The project will contribute to the PL research field by providing a revised PL intervention, a Template for Intervention Description and Replication (TIDieR) checklist, intervention materials, and a protocol that offers a solid empirical and theoretical foundation for a future upscaled effectiveness study to be developed and implemented.

The investigation will be guided by the following research question: is the Y-PATH intervention feasible and acceptable in a Danish school context among pupils in 4^th^ and 5^th^ grade (9-11 years of age)?

**b.** The burden of non-communicable diseases (NCDs) is a rising problem, and the increase in physical inactivity across the world is costly to societies (1). However, the focus on the importance of physical activity (PA) is considerable, yet maintaining sufficient levels of PA is becoming more and more difficult (2,3). Internationally, three in four adolescents, do not currently meet the global recommendations set by the World Health Organization (WHO) (4). Further, a considerable number of Danish children and adolescents do not meet the national physical activity (PA) recommendations (5). It is well-documented that regular PA and decreased sedentary time contribute to the prevention of NCDs as well as NCD risk factors such as e.g. overweight and obesity (4). Despite the well-known benefits of regular PA – promoting mental and physical health across the lifespan – and the evidence that early health behaviors track into adolescence and adulthood (6), studies suggest that PA participation decline notably during adolescence (5,7), with girls being significantly less active than boys (5,8).

The school is considered a key setting to promote PA among children and adolescents. Yet only a few school-based interventions aiming to increase PA, identified small effects, and none, to our knowledge, has shown long-term effects (9,10). Multicomponent interventions that combine two or more intervention approaches such as curricular and non-curricular areas, e.g. physical education lessons, school recess, and after-school leisure time, have shown to be a promising approach to increasing children and adolescents’ PA (11,12). In addition, studies suggest that school-based interventions aiming to increase PA should apply a theoretical basis involving the whole school community comprising e.g. teachers, school managers, and parents (13,14). Further, a crucial factor for the acceptability of a school-based intervention is that it aligns with the schools’ core business and is not a so-called “add-on” to the existing educational curriculum, but an intervention using an “add-in” approach contributing to the core goals of the educational curriculum (15). Besides the small effect sizes identified in school-based interventions aiming to increase PA, it has also been identified that PA is context dependent and thus cannot be treated as a trait.

The concept of physical literacy (PL) has gained popularity worldwide and is a comprehensive multidimensional concept that has been identified as a ‘cause of the causes’. PL consists of the dimensions that lay the foundation for an individual’s capacity and tendency to engage in PA throughout life. Few interventions, however, targeting PL exist on a global scale. In Denmark, the development of theoretically driven and evidence-based PL interventions that aim to increase children and adolescents’ PL are still in their infancy. Yet, a promising theory-based and internationally tested intervention, Youth-Physical Activity Towards Health (Y-PATH), has proven to be effective on children and adolescents’ PA levels, motor skills, and PL in Ireland.

The Y-PATH intervention is a multicomponent holistic school-based intervention based on the theory of PL as well as self-determination theory and the socioecological model (16, 17, 18). The intervention is organized with four core components targeting both pupils, teachers, and parents/guardians, and works through a PE component, a teacher component, a parent/guardian component, and a website component (Figure 1). Y-PATH is facilitated within the principles of ‘add in’ approach to school health promotion as the intervention supports delivery of existing national PE curriculum in schools and does not create additional workload for PE teachers.

The primary aim of Y-PATH is to improve the prerequisites for engagement in lifelong PA, through an improvement of the PL domains. That is, by 1) providing education (improving knowledge and understanding) about the importance of PA for health and the risk of sedentary behavior 2) increasing fundamental movement skills proficiency essential for PA participation, and 3) improving levels of self-efficacy, confidence, motivation, regulation, and empowerment related to PA.

**Figure 1**

The Y-PATH intervention components

**
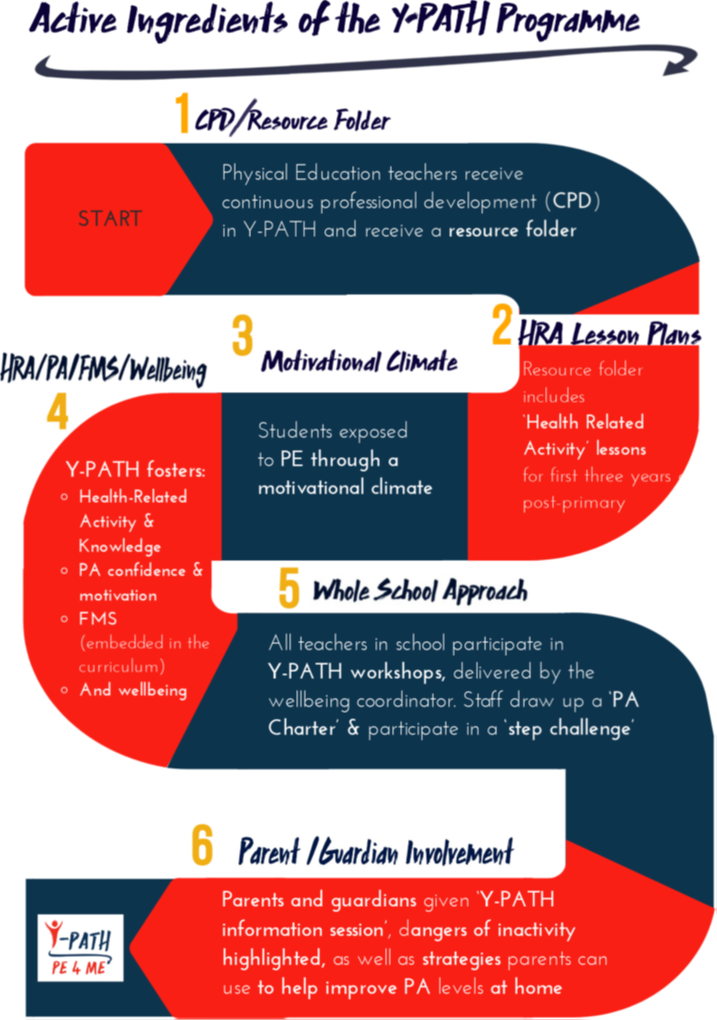
**

**References**

1. Ding D, Lawson KD, Kolbe-Alexander TL, Finkelstein EA, Katzmarzyk PT, van Mechelen W, et al. The economic burden of physical inactivity: a global analysis of major non-communicable diseases. The Lancet. 2016 Sep;388(10051):1311–24.
2. World Health Organization, Regional Office for Europe. Physical activity strategy for the WHO European Region 2016-2025. Copenhagen: World Health Organisation Regional Office for Europe; 2016.
3. World Health Organization. WHO guidelines on physical activity and sedentary behaviour: at a glance. Geneva; 2020.
4. World Health Organization. Global action plan on physical activity 2018–2030: more active people for a healthier world [Internet]. World Health Organization; 2018 [cited 2022 Mar 22]. Available from: <https://apps.who.int/iris/handle/10665/272722>
5. The Ministry of Health. Fysisk aktivitet og stillesiddende adfærd blandt 11-15-årige: National monitorering med objektive målinger (Physical activity and sedentary behavior among 11-15 year olds). Sundhedstyrelsen; 2019.
6. Telama R, Yang X, Viikari J, Välimäki I, Wanne O, Raitakari O. Physical activity from childhood to adulthood: a 21-year tracking study. Am J Prev Med. 2005 Apr;28(3):267–73.
7. Kimm SYS, Glynn NW, Kriska AM, Fitzgerald SL, Aaron DJ, Similo SL, et al. Longitudinal changes in physical activity in a biracial cohort during adolescence. Medicine & Science in Sports & Exercise. 2000 Aug;32(8):1445–54.
8. Riddoch CJ, Bo Andersen L, Wedderkopp N, Harro M, Klasson-Heggebø L, Sardinha LB, et al. Physical Activity Levels and Patterns of 9- and 15-yr-Old European Children. Medicine & Science in Sports & Exercise. 2004 Jan;36(1):86–92.
9. Love R, Adams J, van Sluijs EMF. Are school‐based physical activity interventions effective and equitable? A meta‐analysis of cluster randomized controlled trials with accelerometer‐assessed activity. Obes Rev. 2019 Jun;20(6):859–70.
10. Jones M, Defever E, Letsinger A, Steele J, Mackintosh KA. A mixed-studies systematic review and meta-analysis of school-based interventions to promote physical activity and/or reduce sedentary time in children. Journal of Sport and Health Science. 2020 Jan 1;9(1):3–17.
11. Cohen KE, Morgan PJ, Plotnikoff RC, Callister R, Lubans DR. Physical Activity and Skills Intervention: SCORES Cluster Randomized Controlled Trial. Medicine & Science in Sports & Exercise. 2015 Apr;47(4):765–74.
12. van de Kop JH, van Kernebeek WG, Otten RHJ, Toussaint HM, Verhoeff AP. School-Based Physical Activity Interventions in Prevocational Adolescents: A Systematic Review and Meta-Analyses. Journal of Adolescent Health. 2019 Aug;65(2):185–94.
13. Russ LB, Webster CA, Beets MW, Phillips DS. Systematic Review and Meta-Analysis of Multi-Component Interventions Through Schools to Increase Physical Activity. Journal of Physical Activity and Health. 2015 Oct 1;12(10):1436–46.
14. Sallis JF, McKenzie TL, Conway TL, Elder JP, Prochaska JJ, Brown M, et al. Environmental interventions for eating and physical activity. American Journal of Preventive Medicine. 2003 Apr;24(3):209–17.
15. Bentsen P, Bonde AH, Schneller MB, Danielsen D, Bruselius-Jensen M, Aagaard-Hansen J. Danish ‘add-in’ school-based health promotion: integrating health in curriculum time. Health Promotion International. 2020 Feb 1;35(1):e70–7.
16. Belton, S O’ Brien, W Meegan, S Woods, C Issartel J (2014). Youth Physical Activity Towards Health Evidence and background to the development of the Y PATH physical activity intervention for adolescents BMC Public Health, 14 1 122 https :://doi org/ 10 1186 1471 2458 14 122
17. Belton, S McCarren, A McGrane, B Powell, D Issartel J (2019) The Youth Physical Activity Towards Health (Y PATH) intervention Results of a 24 month cluster randomised controlled trial PLOS ONE, 14 9 e 0221684 https :://doi org/ 10 1371 /journal pone 0221684
18. Belton, S O’Brien, W McGann, J Issartel J (2019) Bright spots physical activity investments that work Youth Physical Activity Towards Health (Y PATH) British Journal of Sports Medicine, 53 4 208 212 https :://doi org/ 10 1136 /bjsports 2018 099745

**c.** Not applicable for the project.

**3. Methods**

**a.** The study builds on a cluster randomized controlled trial (RCT) pilot study and will be carried out in four Danish schools in two different municipalities. The four participating schools will be randomly assigned to either intervention or control conditions using a simple randomization method by drawing a lot. We randomly choose one intervention school and one control school in each municipality. The randomization is done at the school level because the intervention will be facilitated to an entire school – such randomization minimizes the likelihood of unwanted influences on teachers in the control condition, e.g., gaining inspiration from the intervention condition. The school randomized to the intervention condition will receive the intervention, while the control school will be requested to carry out traditional curriculum-based physical education teaching. The control school will, however, be offered the opportunity to receive the intervention the following academic school year. Eligible classes for the study involve 4^th^ and 5^th^ graders, i.e., classes with pupils being 9-11 years of age. Given an approximated mean of 21 pupils per class and two classes per grade, a minimum of 168 pupils will be recruited to receive the intervention.

**b.** Feasibility and acceptability of the intervention will be consistent with guidance proposed by the Medical Research Council (MRC) and The Common Guidelines for Education Research and Development. The evaluation will use a mixed-method approach to assess the primary outcomes – feasibility and acceptability of the Y-PATH intervention. While the quantitative measures will provide knowledge of the intervention effects on pupils’ PL, the qualitative evaluation will provide insights as to why the intervention was effective or not in a Danish school setting. This contributes to potential cultural, context, and age-related alterations. The procedure description is divided in the following: teacher workshop/course, effectiveness, acceptability, and demand experiences, and parents’ questionnaire. An overview of the data collection process can be found in Figure 2. Further, a detailed protocol for the adaptation process can be found online at <https://osf.io/wd5ur/>.

Teacher course: in this study, the intervention teachers will receive a 4-hour face-to-face course and an additional 1,5-hour online course (four modules of 15 to 20 min.) about PL, motivation, and well-being. The PE teachers will receive six detailed PL-based lesson plans which should be delivered in their PE classes. After following the six lesson plans, the teachers will prepare and deliver PL focused PE classes for the rest of the school year. The teachers will be provided with online resources and inspiration materials

Test of effectiveness*:* a design for testing intervention effect will also be tested. This will include administering physical tests and questionnaires. The preliminary effectiveness will be tested by comparing changes in pupils’ PL over time. PL will be assessed with a CAMSA test, pacer test, plank test, and a video-assisted version of the Danish myPL questionnaire and the validated Danish Assessment of Physical Literacy (DAPL) tool, i.e., that was developed in our ongoing project CAPL-2D supported by the TrygFonden (#125640). Furthermore, the children will wear an activity tracker (an accelerometer) for one week twice, i.e., before and after the intervention. Axivity® AX3 accelerometers (23 x 32.5 x 7.6 mm, please see Figure 2) will be mounted with skin tape (with the following brands: Fixomull tape (BSN Medical), adhesive hair-set tape by the investigator to each of the pupils’ left thigh. The effect on pupils’ well-being will also be assessed. This will be assessed using a video-assisted version of KIDSCREEN-27 for 9-11-years-old children as well as a parent-reported version the Strength and Difficulties Questionnaire (SDQ).

Acceptability and demand experiences: the acceptability, including demand and experiences, and the intervention implementation degree, will be evaluated using short bimonthly questionnaires to teachers as well as three observations during the intervention period i.e., one schoolyear. As for the monitoring of the acceptability of the interventions’ materials and resources, the PE teachers will receive a short questionnaire after their weekly PE class – one targeted the six given lesson plans and another one targeted the remaining PE classes. The questions will be related to their PE lessons and their experiences with the focus on PL. As for the three observations, the validated observation tool ‘Multidimensional Motivational Climate Observation System’ will be used in a slightly context-adapted version. Also, the involvement of PL within the PE classes will be observed. The feasibility of the practicality and the recruitment process as well as the costs related to the activities in the project will be assessed within a document log administered by trained research assistants.

Furthermore, interviews will be conducted with pupils, teachers, parents, and school managers after the end of the intervention. Interviews will also be conducted with teachers after the delivery of the six preplanned lesson plans.

Parents’ questionnaires: parents will receive a survey on background information before the start of the intervention as well as an evaluation survey after the intervention.

**Figure 2**

Overview of data collection process


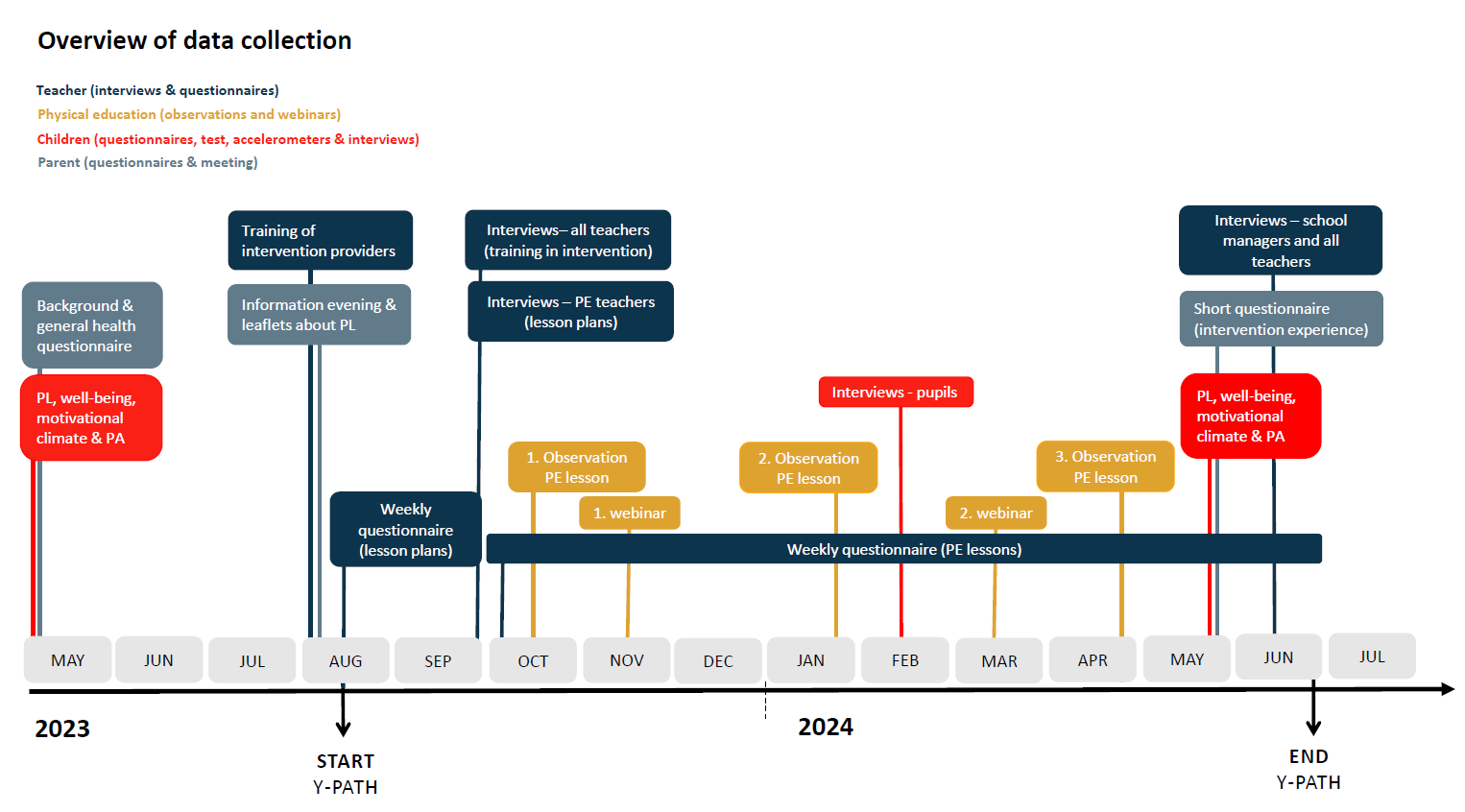


**c.** Not applicable for the project

**4. Statistical considerations**

As this is a pilot study, the analysis will focus on the key parameters necessary for conducting a future trial. Most of the analysis will be descriptive in nature. Descriptive summaries will be generated to detect time and cost used, as well as recruitment process measures. Data on recruitment attrition and questionnaire response rates for pupils will be descriptively summarized and compared between the intervention school and the control school.

Interviews will be transcribed and uploaded into NVivo. Thematic analysis will be carried out for data analysis. This method allows identifying, analyzing, and reporting patterns within the data. Furthermore, it can be used as an essentialist method that reports experiences, meanings, and the reality of participants.

To investigate acceptability of the different intervention components, Qualitative Comparative Analysis (QCA) will be carried out. QCA is a case-oriented method to study complex phenomena originating from the comparative social sciences. This analysis can help identify differences in acceptability (i.e., demand and experiences) between the concerned parties and between the intervention components in order to investigate the mechanism(s). This will help optimizing the revised intervention for a large-scale effectiveness study. All researchers from the project group will validate the analysis, supported by the scientific and national advisory boards.

Preliminary effectiveness of the intervention on PL and well-being will be investigated using mixed models accounting the nested structure of the data. Descriptive statistics (e.g. means, SD) will provide some insight into population characteristics and give an indication of potential changes in mean scores between the two time points (i.e., pre-and postintervention).

**5. Participants**

**a.** Eligible classes for the study involve 4^th^ and 5^th^ graders, i.e., classes with pupils being 9-11 years of age.

A total of four public schools with a minimum of two classes per grade will be recruited and randomized to either the intervention or control condition.

The inclusion criteria are the following:

School/class-level

- Non-special needs municipal school classes
- Classes that are not in other school development or research projects which would most likely influence and bias the results

Pupil-level

- Pupils with parents/guardians who have provided written informed consent, allowing their child to participate

Pupils who have provide an oral assessment

**b.** None in addition to the inclusion criteria provide above.

**6. Risks, Side Effects and Disadvantages**

**a.** No biological measures will be collected. However, there may be risks of which we are not currently aware. Therefore, participants and their parents will be requested to inform any uncomfortable experiences or problems during their participation. In case of recurrent unforeseen ‘side effects’, the data collection procedures or any other parts of the pilot study will be discussed, and adaptions will be implemented to remove these side effects. In addition, the project “Danish Assessment of Physical Literacy” have documented that a small number of children, corresponding to approx. five out of 100, may experience skin irritation when having the Axivity® AX3 accelerometers mounted to the skin with skin tape. However, pupils and parents will be informed to de-mount the accelerometer if this should occur.

**b/c.** Not applicable for the project.

**7. Biological material for use in the project and biobank for future research**

No biobank will be created, thus not appreciable for the project.

**8. Information from patient records**

**a.** Information from patient records will not be collected.

**b.** No data will be collected for research without a written consent and an oral assessment.

**c.** No appreciable.

**9. Processing personal data**

**a.** All data will be stored electronically on a secure drive. The drive will be created by the Center for IT Medico and Technology (CIMT) via a network-file server, as a so-called ‘closed-drive’, which will be logged i.e., CIMT logs who have access to the drive. The drive will be created in accordance with current practice for securely storing personal and sensitive data. Names will not appear on any material, which will instead be affixed to a participant ID-number. Likewise, the names of pupils will not appear in publications of results from this study. The participants are protected by the Act on the Processing of Personal Data and by the Danish Health Care Act, and all personal information is protected by confidentiality. In addition, the project complies with the General Data Protection Regulation and the Data Protection Act. The research project is reviewed and registered in The Capital Region Denmark via PACTIUS (ref. nr. Promoting Pupils’ Physical Literacy (3PL) - P-2022-505).

**b/c/d.** No appreciable.

**10. Economics**

**a.** Center for Clinical Research and Prevention, Copenhagen, Denmark has initiated this feasibility study in collaboration with the Department of Nutrition, Exercise and Sports at Copenhagen University, and the original developers of the irish intervention, i.e., Sarahjane Belton, Wesley O’Brien, and Johann Issartel.

**b.** The Danish foundation TrygFonden has granted the 3PL project 2.120.394 DKK to test the feasibility and acceptability of the previously developed and tested Y-PATH intervention within a Danish school context. The goal being that a revised and adapted ‘3PL intervention’ protocol that aims to increase pupils’ PL, will be ready for effectiveness testing by the end of this project.

In addition, the study will be supported by the original Y-PATH developers Sarahjane Belton, Wesley O’Brien, and Johann Issartel, Professor Mark Tremblay, Director of the Healthy Active Living and Obesity Research Group in Canada (HALO) as well as the consultant, Niels Grinderslev, DGI and assistant professor, Knud Ryom, University of Aarhus.

**c.** See appendix for detailed budget.

**d/e.** Not relevant.

**11. Remuneration**

The participating PE teachers in the two intervention schools will receive 1500DKK as remuneration for the time they use to participate in the workshop before the intervention.

No remuneration will be provided to children or parents.

**12. Recruitment of participants and informed consent**

**a.** The recruitment will proceed through school managers and teachers who will be contacted via e-mail, receiving a recruitment letter and information flyers about the project and participation. Prior to the data collection, parents/guardians of pupils invited to participate in 3PL project will fill out a written informed consent.

**c.** Parents/guardians of pupils invited to participate in the project, will be requested to complete a written consent before data collection. By signing they confirm that they give their child permission to participate. If parents have provided a written consent allowing their child to participate, the child will receive age-appropriate oral information about the project and further be asked to provide an oral assessment. Only when both written consent and oral assessment is obtained, the data will be collected on the respective child.

All data will be stored electronically on a secure drive. The drive will be created by the Center for IT Medico and Technology (CIMT) via a network-file server, as a so-called ‘closed-drive’, which will be logged i.e., CIMT logs who have access to the drive. The drive will be created in accordance with current practice for securely storing personal and sensitive data.

**13. Publications of results**

**a.** The results will be presented at international conferences and published in international peer-reviewed journals – both positive, negative, and in-conclusive results will be published. Prior to the data collection, the project protocol will be registered at ClinicalTrials.gov database.

**14. Ethics**

All data will be anonymized using ID codes and only be analyzed on a group level. Raw data on paper will be stored in a separate locked cabinet only accessible to the primary researchers of the study. Raw data in digital format will be pseudo-anonymized using ID codes and will be stored in a secure project folder at the corporate network drive. Data used for analysis will be of pseudo-anonymized mode. Keys that links ID codes with personally identifiable data will stored in a separate secure project folder at the corporation network drive

Parents/guardians of the children involved in the study will receive written information about the study and will be asked to provide written consent allowing their child to participate. All children will receive age-appropriate oral information and will be asked to provide their consent to participate in the study, if their parents have provided a written consent allowing them to participate. Also, teachers and parents who are invited to participate in interviews will be asked to provide a consent.

**a.**  There are no health risks associated with participation in the research project.

**b.** This research project will lay the foundation for future interventions with the aim of promoting health and well-being as well and preventing non-communicable diseases.

**15. Information about compensation**

Not relevant for the project.
