# Supplementary material for: Feasibility and acceptability of the Promoting Pupils’ Physical Literacy (3PL) intervention and its effectiveness research design: A study protocol
Source: PLoS One. 2024 Jan 5;19(1):e0294916. doi: 10.1371/journal.pone.0294916 (PMC10769070; doi:10.1371/journal.pone.0294916)
Supplement: Peer review history

# Editor report: Decision Letter 0

**DOI:** 10.1371/journal.pone.0294916.r001

**Authors:** Johanna Pruller

13 Nov 2023

Feasibility and acceptability of the Promoting Pupils’ Physical Literacy (3PL) intervention and its effectiveness research design: A study protocol

PONE-D-23-35024

Dear Dr. Toft Amholt,

We’re pleased to inform you that your manuscript has been judged scientifically suitable for publication and will be formally accepted for publication once it meets all outstanding technical requirements.

Within one week, you’ll receive an e-mail detailing the required amendments. When these have been addressed, you’ll receive a formal acceptance letter and your manuscript will be scheduled for publication.

An invoice for payment will follow shortly after the formal acceptance. To ensure an efficient process, please log into Editorial Manager at http://www.editorialmanager.com/pone/, click the 'Update My Information' link at the top of the page, and double check that your user information is up-to-date. If you have any billing related questions, please contact our Author Billing department directly at authorbilling@plos.org.

If your institution or institutions have a press office, please notify them about your upcoming paper to help maximize its impact. If they’ll be preparing press materials, please inform our press team as soon as possible -- no later than 48 hours after receiving the formal acceptance. Your manuscript will remain under strict press embargo until 2 pm Eastern Time on the date of publication. For more information, please contact onepress@plos.org.

Kind regards,

Johanna Pruller, PhD

Associate Editor

PLOS ONE

Journal requirements:

When submitting your revision, we need you to address these additional requirements.

1. Please ensure that your manuscript meets PLOS ONE's style requirements, including those for file naming. The PLOS ONE style templates can be found at 

https://journals.plos.org/plosone/s/file?id=wjVg/PLOSOne_formatting_sample_main_body.pdf and 

https://journals.plos.org/plosone/s/file?id=ba62/PLOSOne_formatting_sample_title_authors_affiliations.pdf

2. Please note that funding information should not appear in any section or other areas of your manuscript. We will only publish funding information present in the Funding Statement section of the online submission form. Please remove any funding-related text from the manuscript.

3. Thank you for stating the following financial disclosure: 

   "This project is funded by TrygFonden (#153181)"

Please state what role the funders took in the study.  If the funders had no role, please state: "The funders had no role in study design, data collection and analysis, decision to publish, or preparation of the manuscript." 

If this statement is not correct you must amend it as needed. 

Please respond by return e-mail so that we can amend your financial disclosure and competing interests on your behalf.

4. PLOS requires an ORCID iD for the corresponding author in Editorial Manager on papers submitted after December 6th, 2016. Please ensure that you have an ORCID iD and that it is validated in Editorial Manager. To do this, go to ‘Update my Information’ (in the upper left-hand corner of the main menu), and click on the Fetch/Validate link next to the ORCID field. This will take you to the ORCID site and allow you to create a new iD or authenticate a pre-existing iD in Editorial Manager. Please see the following video for instructions on linking an ORCID iD to your Editorial Manager account: https://www.youtube.com/watch?v=_xcclfuvtxQ

Please ensure that the text below is included in the Methods section of the manuscript (line 169):

The 3PL study protocol was assessed by the Regional Committee on Health Research Ethics, Capital Region, Denmark (ID: 2106200699). The committee determined that, in accordance with Danish law, the protocol did not qualify for ethics review, as all non-biomedical research projects fall outside the scope of eligibility for ethics assessment. All data and any associated personal information will undergo processing in strict adherence to the Danish Data Protection Act. Prior to enrollment in the study, consent based on written communication was secured from teacher and parents or legal guardians. Oral communication was employed when addressing the children. The data collection processes will be conducted in an engaging and inclusive manner that prioritizes the well-being of the children. Attention will be given to the fact that collecting of questionnaire data may divert time from curricular activities; however, the investigation of motor competence and PA measurement aligns with the existing PE curriculum. Children will be provided with the opportunity to decline PA measurements in the event of potential skin irritation and to opt out of participation in other data collection activities if they find them discomforting. To acknowledge their valuable contributions, teachers and classes will receive separate reimbursements of 200 EUR (1500 DKK) for each participating class.

Reviewers' comments:

# Editor report: Acceptance letter

**DOI:** 10.1371/journal.pone.0294916.r002

**Authors:** Johanna Pruller

29 Dec 2023

PONE-D-23-35024 

PLOS ONE

Dear Dr. Amholt, 

I'm pleased to inform you that your manuscript has been deemed suitable for publication in PLOS ONE. Congratulations! Your manuscript is now being handed over to our production team.

At this stage, our production department will prepare your paper for publication. This includes ensuring the following:

* All references, tables, and figures are properly cited

* All relevant supporting information is included in the manuscript submission,

* There are no issues that prevent the paper from being properly typeset

If revisions are needed, the production department will contact you directly to resolve them. If no revisions are needed, you will receive an email when the publication date has been set. At this time, we do not offer pre-publication proofs to authors during production of the accepted work. Please keep in mind that we are working through a large volume of accepted articles, so please give us a few weeks to review your paper and let you know the next and final steps. 

Lastly, if your institution or institutions have a press office, please let them know about your upcoming paper now to help maximize its impact. If they'll be preparing press materials, please inform our press team within the next 48 hours. Your manuscript will remain under strict press embargo until 2 pm Eastern Time on the date of publication. For more information, please contact onepress@plos.org.

If we can help with anything else, please email us at customercare@plos.org.

Thank you for submitting your work to PLOS ONE and supporting open access. 

Kind regards, 

PLOS ONE Editorial Office Staff

on behalf of

Dr. Johanna Pruller 

Staff Editor

PLOS ONE
